# A Patient Work Lens to Understanding and Supporting Health Care at Home

**DOI:** 10.1093/geroni/igaa057.2913

**Published:** 2020-12-16

**Authors:** Nicole Werner

**Affiliations:** University of Wisconsin-Madison, Madison, Wisconsin, United States

## Abstract

A majority of healthcare today occurs in private homes and is performed by older adults and their informal caregivers. However, only recently have we begun to understand more about the healthcare work of older adults and their caregivers and the home as a healthcare delivery system. Work science allows us to conceptualize the healthcare activities of older adults and their caregivers as a type of health-related work that has been conceptualized under the broad term, patient work. Applying a patient work lens can offer a unique perspective to addressing the persistent and pervasive challenges faced by older adults and their caregivers as they manage their healthcare at home and across healthcare settings. I will describe the historical development of the patient work approach and highlight recent efforts to use a patient work approach to understand and support the work of older adults and their caregivers providing care in the home.

